# Chromosomal alterations in exfoliated urothelial cells from bladder cancer cases and healthy men: a prospective screening study

**DOI:** 10.1186/1471-2407-14-854

**Published:** 2014-11-20

**Authors:** Nadine Bonberg, Beate Pesch, Thomas Behrens, Georg Johnen, Dirk Taeger, Katarzyna Gawrych, Christian Schwentner, Harald Wellhäußer, Matthias Kluckert, Gabriele Leng, Michael Nasterlack, Christoph Oberlinner, Arnulf Stenzl, Thomas Brüning

**Affiliations:** Institute for Prevention and Occupational Medicine of the German Social Accident Insurance, Institute of the Ruhr-Universität Bochum (IPA), Buerkle-de-la-Camp-Platz 1, 44789 Bochum, Germany; Protein Research Unit Ruhr within Europe (PURE), Ruhr-Universität Bochum, Bochum, Germany; Institute of Urology, Eberhard Karls University, Tübingen, Germany; German Social Accident Insurance’s Institution for the Raw Materials and Chemical Industry (BG RCI), Heidelberg, Germany; Department of Health Protection and Occupational Safety, Currenta GmbH & Co. OHG, Leverkusen, Germany; Department of Occupational Medicine and Health Protection, BASF SE, Ludwigshafen, Germany

**Keywords:** Aneuploidy, Bladder cancer, Chromosomal instability, Copy number variation, DNA gain, DNA loss, Fluorescence in situ hybridization, Tetrasomy

## Abstract

**Background:**

Chromosomal instability in exfoliated urothelial cells has been associated with the development of bladder cancer. Here, we analyzed the accumulation of copy number variations (CNVs) using fluorescence in situ hybridization in cancer cases and explored factors associated with the detection of CNVs in tumor-free men.

**Methods:**

The prospective UroScreen study was designed to investigate the performance of UroVysion™ and other tumor tests for the early detection of bladder cancer in chemical workers from 2003–2010. We analyzed a database compiling CNVs of chromosomes 3, 7, and 17 and at 9p21 that were detected in 191,434 exfoliated urothelial cells from 1,595 men. We assessed the accumulation of CNVs in 1,400 cells isolated from serial samples that were collected from 18 cancer cases up to the time of diagnosis. A generalized estimating equation model was applied to evaluate the influence of age, smoking, and urine status on CNVs in cells from tumor-free men.

**Results:**

Tetrasomy of chromosomes 3, 7 and 17, and DNA loss at 9p21 were the most frequently observed forms of CNV. In bladder cancer cases, we observed an accumulation of CNVs that started approximately three years before diagnosis. During the year prior to diagnosis, cells from men with high-grade bladder cancer accumulated more CNVs than those obtained from cases with low-grade cancer (CNV < 2: 7.5% vs. 1.1%, CNV > 2: 16-17% vs. 9-11%). About 1% of cells from tumor-free men showed polysomy of chromosomes 3, 7, or 17 or DNA loss at 9p21. Men aged ≥50 years had 1.3-fold more cells with CNVs than younger men; however, we observed no further age-related accumulation of CNVs in tumor-free men. Significantly more cells with CNVs were detected in samples with low creatinine concentrations.

**Conclusions:**

We found an accumulation of CNVs during the development of bladder cancer starting three years before diagnosis, with more altered cells identified in high-grade tumors. Also, a small fraction of cells with CNVs were exfoliated into urine of tumor-free men, mainly exhibiting tetraploidy or DNA loss at 9p21. Whether these cells are preferentially cleared from the urothelium or are artifacts needs further exploration.

## Background

Chromosomal instability is a common feature of tumor cells, and has been associated with the development of bladder cancer [1, 2]. Alterations in the number of whole chromosomes can lead to chromosomal instability due to segregation errors [3]. For example, tetraploidization may result from a mitotic failure [4], which can occur early in tumorigenesis and foster the accumulation of other forms of genomic instability [5]. Loss and gain of DNA in certain chromosomal segments can cause structural chromosomal instability.

Although the acquisition of genomic alterations has been recognized as a hallmark in the development of cancer, the sequence and role of specific alterations is less clear [6]. DNA amplification in regions hosting oncogenes or the loss of tumor suppressor genes would support the hypothesis that structural chromosomal instability is a driving event. However, aneuploidy has been also found to delay tumor development [7, 8]. Cells with mitotic failure could be eliminated to avoid an accumulation of genomic alterations [9].

Genomic alterations have also been implicated in cellular senescence that accompanies aging [10, 11]. For specific forms of alterations, such as micronuclei, accumulation by age has been demonstrated [12]. Little is known, however, about the accumulation of DNA loss or gain at loci hosting cancer-related driver genes by age or during cancer development [4, 13, 14].

Among the various methods to determine genomic alterations, fluorescence in situ hybridization (FISH) is commonly used to quantify copy number variations (CNVs), represented as DNA loss or gain at the sequences selected for hybridization [15]. UroVysion™ is an approved FISH assay for bladder cancer screening that detects DNA gain and loss of chromosomes 3, 7, and 17 and at the locus 9p21 in exfoliated urothelial cells [16, 17]. The UroScreen study was initiated to validate UroVysion™ and other tumor tests for the early detection of bladder cancer [18–23]. For screening purposes, the extent of genomic alterations is commonly assessed as either positive or negative, and the wealth of CNV data in single cells is usually not documented.

To the best of our knowledge, UroScreen is the first prospective cohort study collecting serial pre-diagnostic samples from asymptomatic subjects, which was used to compile a CNV database with single-cell FISH results. We took advantage of this database to explore the accumulation of DNA gain or loss in serial pre-diagnostic samples from cases and to investigate factors associated with the detection of CNVs in exfoliated urothelial cells from tumor-free men.

## Methods

### Study population and data collection

UroScreen was a prospective screening study aimed to validate UroVysion™ and other tumor tests for the early detection of bladder cancer in 1,609 active or retired chemical workers. The study design and major results on the performance of tumor markers have been described elsewhere [18–20, 22, 23]. Here, we explored CNVs in about 200,000 exfoliated urothelial cells from 6,517 urine samples that were collected from 1,575 cancer-free men and 20 cases during voluntary annual screens between 2003 and 2010. A questionnaire was administered to document smoking history and other information. The ethics committee of the Eberhard Karls University of Tübingen, Germany, approved the study (No. 1/2003 V). All participants gave written informed consent.

### Urine status and UroVysion™ assay

In freshly voided urine, creatinine was determined with the enzymatic test CREA plus® and leukocytes, erythrocytes and other parameters were quantified with Combur^10^ Test® strips (Roche Diagnostics, Mannheim, Germany). Additionally, erythrocytes and leukocytes were detected microscopically in the cell segment as previously described [22]. Urine-status data were documented for 84% of all samples. CNVs were determined with the UroVysion™ assay according to the protocol of the manufacturer (Abbott Laboratories, Abbott Park, IL). In brief, about 25 to 30 morphologically suspicious cells were evaluated in each urine sediment [18]. The UroVysion™ Bladder Cancer Kit is composed of three centromere-specific probes (CEP 3, CEP 7, CEP 17) to capture aneusomy of chromosomes 3, 7, and 17, and the locus-specific indicator probe (LSI) to assess CNV at 9p21. The test was considered to be positive following the manufacturer’s decision rule if ≥4 cells showed polysomy (CNV > 2) of ≥2 chromosomes (3, 7 or 17) or if ≥12 cells had no signal at 9p21 indicating loss of both alleles (CNV = 0).

### Statistical analysis

We described the detailed distributions of DNA gain and loss in terms of CNV for chromosomes 3, 7, 17 and the 9p21 locus in about 200,000 exfoliated urothelial cells. For the cases, we assessed the accumulation of CNVs over time in serial pre-diagnostic samples and the occurrence of CNVs in urine samples collected after diagnosis of bladder cancer. In tumor-free men, we analyzed rate ratios (relative risk, exp) for the influence of age (<50 years as reference), smoking status (never, ever), haematuria, leukocytes and urinary creatinine (<0.5 g/L, ≥0.5 g/L) on the presence of CNVs with generalized estimating equation (GEE) models for repeated measurements [24] with Poisson distribution and log link function. The logarithm of the number of cells analyzed in the samples was used as an offset. Calculations were performed with SAS/STAT and SAS/IML software, version 9.3 (SAS Institute Inc., Cary, NC, USA).

## Results

### Characteristics of the study population of former and active chemical workers

Table 1 describes the study population of 1,575 tumor-free men and 20 cases who developed 21 bladder tumors between 2003 and 2010 (13 high-grade bladder cancers, 5 low-grade bladder cancers, and 3 papillomas). In this prospective study, one case was initially diagnosed with a papilloma, and subsequently with high-grade bladder cancer. The median age of tumor-free men was 62 years (range 27–90 years) in 2010. Cases were diagnosed at a median age of 67 years and included more ever smokers compared to non-diseased men (75% vs. 66%).

**Table 1:** **Characteristics of male active or retired workers with former exposure to aromatic amines**

	Total	Tumor cases ^a^	Cancer cases ^b^	Tumor-free cases
Study participants	1,595	20	18	1,575
Age in years (median and range)				62 (27 – 90)
End of follow-up	62 (27 – 90)	68 (43 – 80)	69 (43 – 80)	
At diagnosis		67 (38 – 78)	67 (38–78)	
Smoking status at baseline [N (%)]^c^				
Never	548 (34.4)	5 (25.0)	4 (22.2)	543 (34.5)
Former	614 (38.5)	12 (60.0)	11 (61.1)	602 (38.4)
Current	430 (27.0)	3 (15.0)	3 (16.7)	427 (27.1)

^a^Tumor cases included three papillomas; one case developed two bladder tumors during UroScreen.

^b^Thirteen high-grade and five low-grade bladder cancers.

^c^Missing for three tumor-free participants.

### Distribution of copy number variations by subtype of bladder tumor

The distribution of CNVs in exfoliated urothelial cells in the year before diagnosis is shown in Table 2, and is stratified by subtype of bladder tumor. Notably, not all subjects participated regularly in this annual voluntary screening program. In exfoliated cells from cases with papilloma, we detected no loss (CNV < 2) at 9p21, and only a small fraction of cells (1%-2%) with tetrasomy (CNV = 4) of chromosomes 3, 7, and 17. The samples from the low-grade bladder cancer cases contained 9%-11% cells with polysomy (CNV > 2) of chromosomes 3, 7, and 17, and 1% cells with loss of both alleles (CNV = 0) at 9p21. More cells with DNA gain (CNV > 2) and DNA loss (CNV < 2) were found in high-grade bladder cancer cases (16%-17% and 7.5%, respectively).

**Table 2:** **Cells with copy number variations (CNVs) of chromosomes 3, 7, 17 and at 9p21 in cases by subtype of bladder tumor in urine samples collected in the year before diagnosis**

CNV			0	1	2	3	4	≥5	Total	Loss (CNV <2)	Gain (CNV >2)
All cases	CEP 3	N (%)	2 (0.3)	0 (0)	554 (87.4)	26 (4.1)	37 (5.8)	15 (2.4)	634 (100)	2 (0.3)	78 (12.3)
	CEP 7	N (%)	2 (0.3)	2 (0.3)	547 (86.3)	26 (4.1)	46 (7.3)	11 (1.7)	634 (100)	4 (0.6)	83 (13.1)
	CEP 17	N (%)	2 (0.3)	3 (0.5)	552 (87.1)	24 (3.8)	35 (5.5)	18 (2.8)	634 (100)	5 (0.8)	77 (12.1)
	LSI 9p21	N (%)	22 (3.5)	8 (1.3)	567 (89.4)	15 (2.4)	21 (3.3)	1 (0.2)	634 (100)	30 (4.7)	37 (5.8)
Papilloma (2 cases, 3 samples)	CEP 3	N (%)	0 (0)	0 (0)	83 (97.6)	0 (0)	2 (2.4)	0 (0)	85 (100)	0 (0)	2 (2.4)
	CEP 7	N (%)	0 (0)	1 (1.2)	83 (97.6)	0 (0)	1 (1.2)	0 (0)	85 (100)	1 (1.2)	1 (1.2)
	CEP 17	N (%)	0 (0)	0 (0)	83 (97.6)	0 (0)	2 (2.4)	0 (0)	85 (100)	0 (0)	2 (2.4)
	LSI 9p21	N (%)	0 (0)	0 (0)	83 (97.6)	1 (1.2)	1 (1.2)	0 (0)	85 (100)	0 (0)	2 (2.4)
Low-grade bladder cancer (5 cases, 6 samples)	CEP 3	N (%)	0 (0)	0 (0)	159 (90.9)	3 (1.7)	7 (4.0)	6 (3.4)	175 (100)	0 (0)	16 (9.1)
	CEP 7	N (%)	0 (0)	0 (0)	156 (89.1)	8 (4.6)	7 (4.0)	4 (2.3)	175 (100)	0 (0)	19 (10.9)
	CEP 17	N (%)	0 (0)	0 (0)	160 (91.4)	7 (4.0)	5 (2.9)	3 (1.7)	175 (100)	0 (0)	15 (8.6)
	LSI 9p21	N (%)	2 (1.1)	0 (0)	163 (93.1)	7 (4.0)	3 (1.7)	0 (0)	175 (100)	2 (1.1)	10 (5.7)
High-grade bladder cancer (9 cases, 12 samples)	CEP 3	N (%)	2 (0.5)	0 (0)	312 (83.4)	23 (6.1)	28 (7.5)	9 (2.4)	374 (100)	2 (0.5)	60 (16.0)
	CEP 7	N (%)	2 (0.5)	1 (0.3)	308 (82.4)	18 (4.8)	38 (10.2)	7 (1.9)	374 (100)	3 (0.8)	63 (16.8)
	CEP 17	N (%)	2 (0.5)	3 (0.8)	309 (82.6)	17 (4.5)	28 (7.5)	15 (4.0)	374 (100)	5 (1.3)	60 (16.0)
	LSI 9p21	N (%)	20 (5.3)	8 (2.1)	321 (85.8)	7 (1.9)	17 (4.5)	1 (0.3)	374 (100)	28 (7.5)	25 (6.7)

### Temporal accumulation of copy number variations of chromosomes 3, 7, and 17 and at the 9p21 locus in exfoliated urothelial cells until diagnosis of bladder cancer

Detailed information about the distribution of CNV in 2,111 exfoliated urothelial cells from cases with bladder cancer is shown in Table 3. We detected only two cells with CNV = 1 of chromosome 17 in urine samples that were collected more than three years before diagnosis. Polysomy of chromosomes 3, 7, and 17 accumulated in up to 14% - 15% of all cells in the year before diagnosis. Tetrasomy (CNV = 4) was the most frequently detected type of polysomy (6% to 8%) followed by trisomy (CNV = 3), which was observed in 4% - 5% of all cells from the cases. Tetrasomy of all three chromosomes (3, 7, and 17) was observed in 3% of the cells in samples collected in the year before diagnosis, compared to only 0.3% in tumor-free men (data not shown). Notably, also few cells could be detected with loss (CNV < 2) of these chromosomes that are prone to gain. Loss of one or both alleles at 9p21 was seen in the cases as early as two to three years before diagnosis. Also, gain at 9p21 accumulated in up to 6% of all cells in the year before diagnosis, compared to the even lower fraction of 4% of cells showing a loss of both alleles at this locus. In screening rounds after diagnosis and treatment, the fraction of cells with polysomy of chromosomes 3, 7, and 17 dropped to 2.3% and DNA loss at 9p21 to 1.7%.

**Table 3:** **Cells with copy number variations (CNVs) of chromosomes 3, 7, 17 and at 9p21 in urines from 18 cases with bladder cancer**

CNV			0	1	2	3	4	≥5	Total	Loss (CNV <2)	Gain (CNV >2)
>3 years before diagnosis	CEP 3	N (%)	0 (0)	0 (0)	350 (100)	0 (0)	0 (0)	0 (0)	350 (100)	0 (0)	0 (0)
	CEP 7	N (%)	0 (0)	0 (0)	350 (100)	0 (0)	0 (0)	0 (0)	350 (100)	0 (0)	0 (0)
	CEP 17	N (%)	0 (0)	2 (0.6)	348 (99.4)	0 (0)	0 (0)	0 (0)	350 (100)	2 (0.6)	0 (0)
	LSI 9p21	N (%)	0 (0)	0 (0)	350 (100)	0 (0)	0 (0)	0 (0)	350 (100)	0 (0)	0 (0)
>2 - 3 years before diagnosis	CEP 3	N (%)	0 (0)	0 (0)	197 (88.3)	9 (4.0)	12 (5.4)	5 (2.2)	223 (100)	0 (0)	26 (11.7)
	CEP 7	N (%)	0 (0)	1 (0.4)	198 (88.8)	10 (4.5)	9 (4.0)	5 (2.2)	223 (100)	1 (0.4)	24 (10.8)
	CEP 17	N (%)	0 (0)	0 (0)	198 (88.8)	8 (3.6)	10 (4.5)	7 (3.1)	223 (100)	0 (0)	25 (11.2)
	LSI 9p21	N (%)	21 (9.4)	3 (1.3)	198 (88.8)	0 (0)	1 (0.4)	0 (0)	223 (100)	24 (10.8)	1 (0.4)
>1 - 2 years before diagnosis	CEP 3	N (%)	0 (0)	0 (0)	256 (92.1)	7 (2.5)	14 (5.0)	1 (0.4)	278 (100)	0 (0)	22 (7.9)
	CEP 7	N (%)	0 (0)	1 (0.4)	257 (92.4)	6 (2.2)	13 (4.7)	1 (0.4)	278 (100)	1 (0.4)	20 (7.2)
	CEP 17	N (%)	0 (0)	3 (1.1)	255 (91.7)	5 (1.8)	14 (5.0)	1 (0.4)	278 (100)	3 (1.1)	20 (7.2)
	LSI 9p21	N (%)	12 (4.3)	5 (1.8)	256 (92.1)	2 (0.7)	3 (1.1)	0 (0)	278 (100)	17 (6.1)	5 (1.8)
≤1 year before diagnosis	CEP 3	N (%)	2 (0.4)	0 (0)	471 (85.8)	26 (4.7)	35 (6.4)	15 (2.7)	549 (100)	2 (0.4)	76 (13.8)
	CEP 7	N (%)	2 (0.4)	1 (0.2)	464 (84.5)	26 (4.7)	45 (8.2)	11 (2.0)	549 (100)	3 (0.5)	82 (14.9)
	CEP 17	N (%)	2 (0.4)	3 (0.5)	469 (85.4)	24 (4.4)	33 (6.0)	18 (3.3)	549 (100)	5 (0.9)	75 (13.7)
	LSI 9p21	N (%)	22 (4.0)	8 (1.5)	484 (88.2)	14 (2.6)	20 (3.6)	1 (0.2)	549 (100)	30 (5.5)	35 (6.4)
After diagnosis	CEP 3	N (%)	0 (0)	0 (0)	695 (97.7)	3 (0.4)	7 (1.0)	6 (0.8)	711 (100)	0 (0)	16 (2.3)
	CEP 7	N (%)	0 (0)	1 (0.1)	694 (97.6)	3 (0.4)	7 (1.0)	6 (0.8)	711 (100)	1 (0.1)	16 (2.3)
	CEP 17	N (%)	0 (0)	1 (0.1)	694 (97.6)	2 (0.3)	5 (0.7)	9 (1.3)	711 (100)	1 (0.1)	16 (2.3)
	LSI 9p21	N (%)	10 (1.4)	2 (0.3)	695 (97.7)	1 (0.1)	3 (0.4)	0 (0)	711 (100)	12 (1.7)	4 (0.6)

### Factors influencing the detection of copy number variations of chromosomes 3, 7, and 17 and at the 9p21 locus in exfoliated urothelial cells from tumor-free men

Table 4 shows the distribution of CNVs assessed with CEP 3, CEP 7, CEP 17, and LSI 9p21 in 188,911 exfoliated urothelial cells from tumor-free men. Overall, most cells were diploid. We observed DNA loss (CNV < 2) at 9p21 in 1.0% of all cells, and CNV > 2 at all four loci in about 1% of all cells. CNV = 4 was the most common type of DNA gain, found in 0.5% to 0.7% of all cells. We observed slightly more CNVs in cells from men ≥50 years of age compared to younger participants. For example, the fraction of cells with polysomy of chromosome 7 was 0.9% in men aged <50 years and 1.2% in men ≥50 years of age. The corresponding figures for age-related DNA loss (CNV < 2) at 9p21 were 0.8% and 1.0%, respectively. There was a small difference in DNA gain between never and ever smokers. The fraction of cells with polysomy of chromosome 7 was 0.9% in never smokers and 1.1% in ever smokers. No such difference was observed for DNA loss at 9p21 (1.0% in both never and ever smokers). Although ever smokers aged ≥50 years had slightly more cells with CNVs than never smokers aged <50 years, the small difference (~0.5%) was not significant (data not shown).

**Table 4:** **Cells with copy number variations (CNVs) of chromosomes 3, 7, and 17 and at 9p21 in urines from 1,575 tumor-free men**

CNV	Locus		0	1	2	3	4	≥5	Total	Loss (CNV <2)	Gain (CNV >2)
**Total**	CEP 3	N (%)	130 (0.1)	260 (0.1)	186508 (98.7)	549 (0.3)	1313 (0.7)	151 (0.1)	188911 (100)	390 (0.2)	2013 (1.1)
	CEP 7	N (%)	5 (0)	327 (0.2)	186430 (98.7)	702 (0.4)	1317 (0.7)	130 (0.1)	188911 (100)	332 (0.2)	2149 (1.1)
	CEP 17	N (%)	114 (0.1)	907 (0.5)	186242 (98.6)	657 (0.3)	904 (0.5)	87 (0.0)	188911 (100)	1021 (0.5)	1648 (0.9)
	LSI 9p21	N (%)	736 (0.4)	1089 (0.6)	185417 (98.2)	431 (0.2)	1156 (0.6)	82 (0.0)	188911 (100)	1825 (1.0)	1669 (0.9)
**Age (years)** ^**a**^											
<50	CEP 3	N (%)	32 (0.1)	62 (0.1)	47873 (98.9)	136 (0.3)	251 (0.5)	29 (0.1)	48383 (100)	94 (0.2)	416 (0.9)
	CEP 7	N (%)	1 (0.0)	80 (0.2)	47861 (98.9)	151 (0.3)	257 (0.5)	33 (0.1)	48383 (100)	81 (0.2)	441 (0.9)
	CEP 17	N (%)	15 (0.0)	192 (0.4)	47854 (98.9)	137 (0.3)	167 (0.3)	18 (0.0)	48383 (100)	207 (0.4)	322 (0.7)
	LSI 9p21	N (%)	162 (0.3)	230 (0.5)	47638 (98.5)	77 (0.2)	248 (0.5)	28 (0.1)	48383 (100)	392 (0.8)	353 (0.7)
≥50	CEP 3	N (%)	98 (0.1)	198 (0.1)	138635 (98.7)	413 (0.3)	1062 (0.8)	122 (0.1)	140528 (100)	296 (0.2)	1597 (1.1)
	CEP 7	N (%)	4 (0.0)	247 (0.2)	138569 (98.6)	551 (0.4)	1060 (0.8)	97 (0.1)	140528 (100)	251 (0.2)	1708 (1.2)
	CEP 17	N (%)	99 (0.1)	715 (0.5)	138388 (98.5)	520 (0.4)	737 (0.5)	69 (0.0)	140528 (100)	814 (0.6)	1326 (0.9)
	LSI 9p21	N (%)	574 (0.4)	859 (0.6)	137779 (98.0)	354 (0.3)	908 (0.6)	54 (0.0)	140528 (100)	1433 (1.0)	1316 (0.9)
**Smoking**											
Never	CEP 3	N (%)	37 (0.1)	91 (0.2)	51871 (98.8)	140 (0.3)	320 (0.6)	33 (0.1)	52492 (100)	128 (0.2)	493 (0.9)
	CEP 7	N (%)	1 (0.0)	92 (0.2)	51865 (98.8)	198 (0.4)	312 (0.6)	24 (0.0)	52492 (100)	93 (0.2)	534 (1.0)
	CEP 17	N (%)	31 (0.1)	258 (0.5)	51808 (98.7)	147 (0.3)	230 (0.4)	18 (0.0)	52492 (100)	289 (0.6)	395 (0.8)
	LSI 9p21	N (%)	199 (0.4)	307 (0.6)	51556 (98.2)	106 (0.2)	302 (0.6)	22 (0.0)	52492 (100)	506 (1.0)	430 (0.8)
Ever	CEP 3	N (%)	93 (0.1)	169 (0.1)	134637 (98.7)	409 (0.3)	993 (0.7)	118 (0.1)	136419 (100)	262 (0.2)	1520 (1.1)
	CEP 7	N (%)	4 (0.0)	235 (0.2)	134565 (98.6)	504 (0.4)	1005 (0.7)	106 (0.1)	136419 (100)	239 (0.2)	1615 (1.2)
	CEP 17	N (%)	83 (0.1)	649 (0.5)	134434 (98.5)	510 (0.4)	674 (0.5)	69 (0.1)	136419 (100)	732 (0.5)	1253 (0.9)
	LSI 9p21	N (%)	537 (0.4)	782 (0.6)	133861 (98.1)	325 (0.2)	854 (0.6)	60 (0.0)	136419 (100)	1319 (1.0)	1239 (0.9)
**Creatinine (g/L)** ^**a**^											
<0.5	CEP 3	N (%)	25 (0.1)	42 (0.2)	24377 (98.0)	129 (0.5)	254 (1.0)	39 (0.2)	24866 (100)	67 (0.3)	422 (1.7)
	CEP 7	N (%)	1 (0.0)	43 (0.2)	24361 (98.0)	142 (0.6)	273 (1.1)	46 (0.2)	24866 (100)	44 (0.2)	461 (1.9)
	CEP 17	N (%)	21 (0.1)	159 (0.6)	24361 (98.0)	142 (0.6)	155 (0.6)	28 (0.1)	24866 (100)	180 (0.7)	325 (1.3)
	LSI 9p21	N (%)	135 (0.5)	166 (0.7)	24205 (97.3)	88 (0.4)	248 (1.0)	24 (0.1)	24866 (100)	301 (1.2)	360 (1.4)
≥0.5	CEP 3	N (%)	56 (0.0)	166 (0.1)	134228 (98.9)	356 (0.3)	865 (0.6)	78 (0.1)	135749 (100)	222 (0.2)	1299 (1.0)
	CEP 7	N (%)	4 (0.0)	227 (0.2)	134144 (98.8)	439 (0.3)	864 (0.6)	71 (0.1)	135749 (100)	231 (0.2)	1374 (1.0)
	CEP 17	N (%)	60 (0.0)	572 (0.4)	134036 (98.7)	424 (0.3)	614 (0.5)	43 (0.0)	135749 (100)	632 (0.5)	1081 (0.8)
	LSI 9p21	N (%)	456 (0.3)	708 (0.5)	133478 (98.3)	285 (0.2)	770 (0.6)	52 (0.0)	135749 (100)	1164 (0.9)	1107 (0.8)

^a^Samples from one person collected at different time points could be allocated into two different groups, due to the longitudinal design of the UroScreen study.

In addition to the individual differences of each subject, urine density also influenced the detection of CNVs in exfoliated urothelial cells. Cells in the voided urine that had low creatinine content (<0.5 g/L) contained increased amount of both DNA gain and loss, compared with cells exfoliated into urine with creatinine concentration ≥0.5 g/L. The corresponding figures for cells with polysomy of chromosome 7 were 1.9% vs. 1.0%.

Table 5 shows the effect estimates from a GEE model that examined factors which may influence the incidence of CNVs in cells from tumor-free men. A low urinary creatinine concentration had the strongest effect on detecting deviations from the diploid karyotype that was assessed as polysomy in at least one of the chromosomes 3, 7, and 17 or a DNA loss at the 9p21 locus (exp  = 1.58 (95% confidence interval (CI) 1.34 – 1.86) for creatinine <0.5 g/L as compared to creatinine ≥0.5 g/L). This effect was also significant for all single probes. We further confirmed the age effect after adjustment for creatinine and other factors. More cells with CNVs could be observed in the urine from men aged ≥50 years as compared with younger men. However, there was no further accumulation of CNVs by age (50 – <60 years: exp  = 1.30, 60 – <70 years: exp  = 1.20, ≥70 years: exp  = 1.31). The effect of age on DNA loss was less pronounced than on DNA gain. The influence of ever smoking on CNVs was only marginal, with exp  = 1.14 (95% CI 0.97 – 1.32) for all probes, only achieving significance for polysomy of chromosome 17 (exp  = 1.33, 95% CI 1.05 – 1.70). The presence of urinary leukocytes or erythrocytes seemed to impair the detection of CNVs in urothelial cells, which was statistically significant for detecting polysomy of chromosome 17.

**Table 5:** **Potential predictors of cells with copy number variations (CNVs) in urine from 1,575 tumor-free men**

			CNV > 2 Chromosome 3	CNV > 2 Chromosome 7	CNV > 2 Chromosome 17	CNV < 2 9p21	CNV > 2 of chromosome 3, 7 or 17 or CNV < 2 at 9p21
		Reference	exp (95% CI) ^a^	exp (95% CI) ^a^	exp (95% CI) ^a^	exp (95% CI) ^a^	exp (95% CI) ^a^
**Creatinine**	< 0.5 g/L	≥ 0.5 g/L	1.69 (1.31 – 2.18)	1.73 (1.35 – 2.23)	1.53 (1.18 – 1.97)	1.40 (1.12 – 1.74)	1.58 (1.34 – 1.86)
**Leukocytes** ^**b**^	Present	None or traces^b^	0.95 (0.58 – 1.56)	0.94 (0.58 – 1.52)	0.88 (0.52 – 1.48)	0.78 (0.52 – 1.16)	0.85 (0.62 – 1.17)
**Haematuria** ^**b**^	Present	None or traces^b^	0.78 (0.59 – 1.04)	0.78 (0.59 – 1.02)	0.75 (0.56 – 0.99)	0.99 (0.80 – 1.24)	0.89 (0.75 – 1.05)
**Age (years)**	50 – <60	<50	1.54 (1.11 – 2.13)	1.57 (1.14 – 2.15)	1.50 (1.10 – 2.06)	1.22 (0.95 – 1.56)	1.30 (1.06 – 1.59)
	60 – <70	<50	1.24 (0.92 – 1.67)	1.26 (0.95 – 1.68)	1.37 (1.02 – 1.82)	1.21 (0.97 – 1.50)	1.20 (1.00 – 1.43)
	≥ 70	<50	1.45 (1.04 – 2.03)	1.55 (1.11 – 2.16)	1.74 (1.23 – 2.45)	1.24 (0.93 – 1.66)	1.31 (1.06 – 1.62)
**Smoking**	Ever	Never	1.26 (0.98 – 1.62)	1.27 (0.99 – 1.64)	1.33 (1.05 – 1.70)	1.11 (0.92 – 1.34)	1.14 (0.97 – 1.32)

^a^Effect estimates as factors from a generalized estimating equations model with built-in Poisson distribution.

^b^None or traces if ≤5 cells per visual field microscopically and dipstick testing negative or <5 leukocytes.

## Discussion

### DNA copy number variations in exfoliated urothelial cells from bladder tumor cases and tumor-free men

Genomic instability is a hallmark of cancer [25, 26], where aneuploidy is a common feature of tumorigenesis (e.g. [4, 27]). We assessed CNVs as numerical changes of the chromosomes 3, 7, and 17, and of the 9p21 locus in nearly 200,000 exfoliated urothelial cells from 1,595 male participants of the UroScreen study. In previous analyses, we qualitatively assessed the extent of CNVs as either a positive or negative result of the UroVysion™ test that is normally used for bladder cancer screening [18, 23]. Here, we quantitatively analyzed CNVs in order to explore the accumulation of CNVs during the development of bladder tumors in serial pre-diagnostic samples collected from cases. CNVs were rare three or more years before the diagnosis of bladder cancer and in exfoliated cells from papilloma cases. Loss of both alleles at 9p21 was detected as an early event but without further accumulation. In contrast, the fraction of cells with CNV > 2 continued to increase up to the time of diagnosis, with more cells affected in cases with high-grade than low-grade bladder cancer. The extent of DNA gain but not of loss produced a positive result in the UroVysion^TM^ test, usually not earlier than in the year before diagnosis [18, 19]. Also, urine sediments from tumor-free men contained about 1% cells with CNVs. We found slightly more affected cells in men ≥50 years old, and considerably more cells with CNVs in urine samples where the creatinine concentration was <0.5 g/L.

The aneuploidy status of late-stage tumors provides little information about the temporal accumulation of genomic alterations during tumor development [10]. An inadequate supply of human tissues from early disease stages due to the lack of serial pre-diagnostic samples from prospective studies is a severe limitation for cancer research [28, 29]. The main advantage of UroScreen is the prospective design and a database compiling CNVs in all single cells that were measured with UroVysion™. This allowed us to assess the accumulation of CNVs until diagnosis of bladder tumor. A general limitation is the low incidence of bladder tumors in asymptomatic subjects [23, 30]. Further, the preference of morphologically aberrant cells for assessing CNVs according to the protocol of the manufacturer may overestimate the extent of genomic alterations [19, 23].

### Tetrasomy in the development of bladder cancer

Cancer is characterized by uncontrolled cell growth, where mitotic failure may occur, leading to tetraploidization as an early event in tumorigenesis [5]. Tetraploidy may therefore indicate a higher proliferation rate as well as failure of cytokinesis [5]. Affected cells do not accurately segregate whole chromosomes. The UroVysion™ assay allows the determination of aneusomy of chromosomes 3, 7, and 17 with three probes designed for centromere-specific sequences. Tetrasomy was the most commonly observed CNV in cases as well as in tumor-free men from UroScreen. In cases, we observed more cells with tetrasomy starting three years before diagnosis, compared to earlier samples or tumor-free men. The fraction of exfoliated cells with CNV = 4 increased by the degree of malignancy up to the time of diagnosis, reaching 2.4% in papillomas, 4% in low-grade bladder cancer, and 10% in high-grade bladder cancer. Notably, tetrasomy of these three chromosomes occurred in parallel in cells isolated from cases as well as from tumor-free men, indicating tetraploidy.

### Aneusomy of chromosomes 3, 7 and 17 during cancer development

Tetraploid cells are prone to additional numerical and structural aberrations commonly defined as aneuploidy [27], and other forms of genomic instability [5, 31, 32]. The fraction of cells with trisomy of chromosomes 3, 7, or 17 increased from 0.3%-0.4% in tumor-free men, and up to 4%-5% in cases with bladder cancer when approaching time of diagnosis. Also, CNV ≥5 occurred in cases with a maximum of 10 copy numbers detected in exfoliated cells. The amount of exfoliated cells that accumulated gains was higher in high-grade bladder cancer compared to low-grade bladder cancer, whereas aneusomy was not detected in cells from any of the papilloma cases. Monosomy was rare and showed no clear increase during cancer development. Former statistical analyses on the UroVysion™ data, which were performed to improve the tumor test, demonstrated that CNVs of chromosomes 3, 7, and 17 were strongly correlated [19].

### Deletion at 9p21 during cancer development

Besides alterations in the number of chromosomes, structural aberrations like the deletions of DNA sequences encoding tumor suppressors or other cancer-related genes may also occur during the development of cancer. For example, loss of cyclin-dependent kinase inhibitor 1B (*CDKN1B*) has been described in 23% of prostate cancers [33]. The 9p21 locus encompasses cyclin-dependent kinase inhibitor 2A (*CDKN2A*), encoding p16 and p14 [14]. Loss of 9p21 has been observed in bladder cancer and other malignancies (e.g., [34, 35]) and is considered an early step in the progression of urinary bladder carcinogenesis [36, 37]. We confirmed an increased rate of homozygous deletions early in the development of bladder cancer. There was rarely a loss of alleles in papilloma cases. Cases with high-grade bladder cancer exhibited a larger percentage of cells with loss at 9p21 compared to those with low-grade bladder cancer. In line with another study [38], the extent of DNA loss at 9p21 observed in UroScreen was not sufficient to achieve positivity of the UroVysion test [19]. Weak signals of this probe may lead to false-negative results [38].

Whereas malignancy has been associated with a complete loss at 9p21, the impact of losing one allele is less clear [39]. We observed 1.5% cells with CNV = 1 compared to 4% with a complete loss of 9p21 in cases prior to the diagnosis of bladder cancer. The corresponding figures were 0.6% and 0.4% in tumor-free men, respectively. A loss of one allele may be combined with a mutation or hypermethylation of the other allele and, therefore, a complete loss of *CDKN2A* function cannot be captured by determining CNV alone [40]. However, point mutations or hypermethylation at this locus may not be frequent in bladder cancer [1].

### The distribution of DNA copy number variations in exfoliated urothelial cells from tumor-free men

To our knowledge, we explored the largest collection of FISH data in tumor-free subjects, although distributions of similar datasets have been previously published [41, 42]. More than 40% of all tumor-free men in UroScreen had at least one cell with at least one CNV in repeatedly collected urine samples. In line with a previous report [41], we detected about 1% of cells with CNV > 2, mainly as tetrasomy. One explanation could be that some cells were in S or G2 phase of the cell cycle [41]. We detected a similar fraction of 1% of cells with CNV < 2 at 9p21. However, the observed loss of DNA at this locus was lower compared to other reports [41, 42]. A small number of cells with aneuploidy does not necessarily indicate cancer development as previously suggested [41]. In addition, assay-specific problems should be taken into account. For example, two overlapping signals may appear as one, or the hybridization of the probes was not efficient [41].

### The influence of age and smoking on the occurrence of DNA copy number variations

Age is the strongest risk factor for the development of cancer, with a steep rise of incidence after the age of 50 years. Aging may be accompanied by random alterations of the genome that could accumulate and increase the vulnerability for cancer-specific alterations [43]. Studies of the mitotic checkpoint regulator BubR1 demonstrated the association between age and aneuploidization [44]. We observed about 30% more cells with polysomy in participants aged ≥50 years compared to younger men. However, little is known about the age-related accumulation of CNVs in sequences encoding for tumor suppressors or oncogenes in humans. Cells with DNA loss at 9p21 seem not to accumulate with increasing age. It therefore appears plausible that the amplification or loss of cancer-related genes has a higher probability of leading to cancer than a “silent” alteration, which can accumulate during aging.

Although smoking is an established risk factor for developing cancer of the bladder [45], little is known about the contribution of smoking to the development of chromosomal instability [13, 14]. We observed slightly more cells with CNVs in tumor-free men who reported having ever smoked, but this was of marginal significance. A more precise assessment of smoking behavior would be necessary to refine a potential effect of smoking on the development of genomic alterations.

### Exfoliation of urothelial cells with copy number variations – also a clearance mechanism?

During the assessment of the performance of the UroVysion™ test, we observed that considerably more aberrant cells were detected in urine samples from cases with a low creatinine content [22]. Here, we could demonstrate a similar and strong effect in this large group of tumor-free subjects. Approximately twofold more cells with CNVs were detected in diluted urine with creatinine concentrations <0.5 g/L. The interpretation of this effect is challenging. Assay-specific problems may occur in samples with higher cellularity [41]. Furthermore, low osmolarity in diluted urine may impair the hybridization efficiency of UroVysion™ probes. Another hypothesis is that urothelial cells with mitotic failure are exfoliated at a higher rate as a clearance mechanism in order to avoid the accumulation of genomic alterations in the target tissue [9].

The instillation of urinary tract infections is increasingly applied as a form of effective immunotherapy to better eliminate cancer cells from the urothelium [46]. Cell death following aberrant mitosis or other damaging events may be initiated to avoid persistence of genomic instability [7]. Apoptosis and other senescence mechanisms can “clear” the target tissue from genomic alterations. The sensitivity of cancer cells to therapeutic agents might stimulate this clearance. We observed that in cases after diagnosis, the fraction of cells with CVNs was still twice the average level to those measured in tumor-free men. However, the exfoliation of damaged cells may also be explained by the high recurrence rate of bladder tumors indicating the development of a subsequent tumor.

## Conclusions

We observed an accumulation of CNVs in exfoliated urothelial cells assessed with polysomy of chromosomes 3, 7, and 17 and loss at 9p21 during the development of bladder cancer in serial pre-diagnostic samples from cases. High-grade bladder cancer was associated with more DNA loss and gain than low-grade bladder cancer, whereas CNVs were rarely found in exfoliated cells from papilloma cases. CNVs were higher in cases as early as three years before diagnosis compared with tumor-free men, but the extent required for a positive UroVysion™ test is usually achieved no earlier than a year before diagnosis. This should be considered when using the test for the early detection of bladder cancer. We also observed elevated CNVs in screening rounds after diagnosis. Whether this indicates a recurrent bladder cancer or the clearance of the target tissue from damaged cells fostered by immunotherapy remains to be investigated.

## Abbreviations

CNV: Copy number variation
FISH: Fluorescence in situ hybridization
CEP: Centromere-specific probe
LSI: Locus-specific indicator probe
CDKN1B: Cyclin-dependent kinase inhibitor 1B
CDKN2A: Cyclin-dependent kinase inhibitor 2A
CI: Confidence interval.

## References

[1] Florl AR, Schulz WA (2008). Chromosomal instability in bladder cancer. Arch Toxicol.

[2] Lauss M, Aine M, Sjödahl G, Veerla S, Patschan O, Gudjonsson S, Chebil G, Lövgren K, Fernö M, Månsson W, Liedberg F, Ringnér M, Lindgren D, Höglund M (2012). DNA methylation analyses of urothelial carcinoma reveal distinct epigenetic subtypes and an association between gene copy number and methylation status. Epigenetics.

[3] Duesberg P, Li R (2003). Multistep carcinogenesis: a chain reaction of aneuploidizations. Cell Cycle.

[4] Ricke RM, van Ree JH, van Deursen JM (2008). Whole chromosome instability and cancer: a complex relationship. Trends Genet.

[5] Lv L, Zhang T, Yi Q, Huang Y, Wang Z, Hou H, Zhang H, Zheng W, Hao Q, Guo Z, Cooke HJ, Shi Q (2012). Tetraploid cells from cytokinesis failure induce aneuploidy and spontaneous transformation of mouse ovarian surface epithelial cells. Cell Cycle.

[6] Duesberg P, Li R, Fabarius A, Hehlmann R (2006). Aneuploidy and cancer: from correlation to causation. Contrib Microbiol.

[7] Vitale I, Galluzzi L, Castedo M, Kroemer G (2011). Mitotic catastrophe: a mechanism for avoiding genomic instability. Nat Rev Mol Cell Biol.

[8] Weaver BA, Cleveland DW (2009). The role of aneuploidy in promoting and suppressing tumors. J Cell Biol.

[9] Weaver BA, Cleveland DW (2007). Aneuploidy: instigator and inhibitor of tumorigenesis. Cancer Res.

[10] Ricke RM, van Deursen JM (2013). Aneuploidy in health, disease, and aging. J Cell Biol.

[11] Vijg J, Suh Y (2013). Genome instability and aging. Annu Rev Physiol.

[12] Fenech M, Holland N, Zeiger E, Chang WP, Burgaz S, Thomas P, Bolognesi C, Knasmueller S, Kirsch-Volders M, Bonassi S (2011). The HUMN and HUMNxL international collaboration projects on human micronucleus assays in lymphocytes and buccal cells–past, present and future. Mutagenesis.

[13] Gabriel U, Giehl M, Haass W, Trojan L, Michel MS, Hofmann WK, Seifarth W, Fabarius A (2010). Urine from current smokers induces centrosome aberrations and spindle defects in vitro in nonmalignant human cell lines. Cancer Genet Cytogenet.

[14] Lenz P, Pfeiffer R, Baris D, Schned AR, Takikita M, Poscablo MC, Schwenn M, Johnson A, Jones M, Kida M, Cantor KP, Rothman N, Silverman DT, Hewitt SM, Moore LE (2012). Cell-cycle control in urothelial carcinoma: large-scale tissue array analysis of tumor tissue from Maine and Vermont. Cancer Epidemiol Biomarkers Prev.

[15] Furney SJ, Gundem G, Lopez-Bigas N (2012). Oncogenomics methods and resources. Cold Spring Harb Protoc.

[16] **UroVysion** http://www.accessdata.fda.gov/cdrh_docs/pdf3/k033982.pdf

[17] Habuchi T, Marberger M, Droller MJ, Hemstreet GP, Grossman HB, Schalken JA, Schmitz-Dräger BJ, Murphy WM, Bono AV, Goebell P, Getzenberg RH, Hautmann SH, Messing E, Fradet Y, Lokeshwar VB (2005). Prognostic markers for bladder cancer: International Consensus Panel on bladder tumor markers. Urology.

[18] Banek S, Schwentner C, Täger D, Pesch B, Nasterlack M, Leng G, Gawrych K, Bonberg N, Johnen G, Kluckert M, Gakis G, Todenhöfer T, Hennenlotter J, Brüning T, Stenzl A, UroScreen Study Group: **Prospective evaluation of fluorescence-in-situ-hybridization to detect bladder cancer: Results from the UroScreen-study.** *Urol Oncol* doi:101016/jurolonc 2012 04 015 2012 10.1016/j.urolonc.2012.04.015 22621963

[19] Bonberg N, Taeger D, Gawrych K, Johnen G, Banek S, Schwentner C, Sievert KD, Wellhäußer H, Kluckert M, Leng G, Nasterlack M, Stenzl A, Behrens T, Brüning T, Pesch B, UroScreen Study Group: **Chromosomal instability and bladder cancer - the UroVysion™ test in the UroScreen study.** *BJU Int* doi:101111/j1464–410X201211666x2013 10.1111/j.1464-410X.2012.11666.x 23350736

[20] Huber S, Schwentner C, Taeger D, Pesch B, Nasterlack M, Leng G, Mayer T, Gawrych K, Bonberg N, Pelster M, Johnen G, Bontrup H, Wellhäusser H, Bierfreund HG, Wiens C, Bayer C, Eberle F, Scheuermann B, Kluckert M, Feil G, Brüning T, Stenzl A, UroScreen Study Group (2012). Nuclear matrix protein-22: a prospective evaluation in a population at risk for bladder cancer: results from the UroScreen study. BJU Int.

[21] Johnen G, Gawrych K, Bontrup H, Pesch B, Taeger D, Banek S, Kluckert M, Wellhäußer H, Eberle F, Nasterlack M, Leng G, Stenzl A, Brüning T, UroScreen Study Group (2012). Performance of survivin mRNA as a biomarker for bladder cancer in the prospective study UroScreen. PLoS One.

[22] Pesch B, Nasterlack M, Eberle F, Bonberg N, Taeger D, Leng G, Feil G, Johnen G, Ickstadt K, Kluckert M, Wellhäusser H, Stenzl A, Brüning T, UroScreen Group (2011). The role of haematuria in bladder cancer screening among men with former occupational exposure to aromatic amines. BJU Int.

[23] Pesch B, Taeger D, Johnen G, Gawrych K, Bonberg N, Schwentner C, Wellhäusser H, Kluckert M, Leng G, Nasterlack M, Lotan Y, Stenzl A, Brüning T, UroScreen Study Group (2013). Screening for bladder cancer with urinary tumor markers in chemical workers with exposure to aromatic amines. Int Arch Occup Environ Health.

[24] Zeger SL, Liang KY (1986). Longitudinal data analysis for discrete and continuous outcomes. Biometrics.

[25] Hanahan D, Weinberg RA (2000). The hallmarks of cancer. Cell.

[26] Hanahan D, Weinberg RA (2011). Hallmarks of cancer: the next generation. Cell.

[27] Rajagopalan H, Lengauer C (2004). Aneuploidy and cancer. Nature.

[28] Brenner DE, Normolle DP (2007). Biomarkers for cancer risk, early detection, and prognosis: the validation conundrum. Cancer Epidemiol Biomarkers Prev.

[29] Pesch B, Brüning T, Johnen G, Casjens S, Bonberg N, Taeger D, Müller A, Weber DG, Behrens T (2014). Biomarker research with prospective study designs for the early detection of cancer. Biochim Biophys Acta.

[30] Lotan Y, Shariat SF, Schmitz-Dräger BJ, Sanchez-Carbayo M, Jankevicius F, Racioppi M, Minner SJ, Stöhr B, Bassi PF, Grossman HB (2010). Considerations on implementing diagnostic markers into clinical decision making in bladder cancer. Urol Oncol.

[31] Vitale I, Galluzzi L, Senovilla L, Criollo A, Jemaà M, Castedo M, Kroemer G (2011). Illicit survival of cancer cells during polyploidization and depolyploidization. Cell Death Differ.

[32] Cimini D, Degrassi F (2005). Aneuploidy: a matter of bad connections. Trends Cell Biol.

[33] Kibel AS, Faith DA, Bova GS, Isaacs WB (2000). Loss of heterozygosity at 12P12-13 in primary and metastatic prostate adenocarcinoma. J Urol.

[34] Czarnecka KH, Migdalska-Sęk M, Antczak A, Pastuszak-Lewandoska D, Kordiak J, Nawrot E, Domańska D, Kaleta D, Górski P, Brzeziańska EB (2013). Allelic imbalance in 1p, 7q, 9p, 11p, 12q and 16q regions in non-small cell lung carcinoma and its clinical association: a pilot study. Mol Biol Rep.

[35] Chin L, Garraway LA, Fisher DE (2006). Malignant melanoma: genetics and therapeutics in the genomic era. Genes Dev.

[36] Czerniak B, Chaturvedi V, Li L, Hodges S, Johnston D, Roy JY, Luthra R, Logothetis C, Von Eschenbach AC, Grossman HB, Benedict WF, Batsakis JG (1999). Superimposed histologic and genetic mapping of chromosome 9 in progression of human urinary bladder neoplasia: implications for a genetic model of multistep urothelial carcinogenesis and early detection of urinary bladder cancer. Oncogene.

[37] Louhelainen J, Wijkstrom H, Hemminki K (2000). Initiation-development modelling of allelic losses on chromosome 9 in multifocal bladder cancer. Eur J Cancer.

[38] Köhler CU, Martin L, Bonberg N, Behrens T, Deix T, Braun K, Noldus J, Jöckel KH, Erbel R, Sommerer F, Tannapfel A, Harth V, Käfferlein HU, Brüning T (2014). Automated quantification of FISH signals in urinary cells enables the assessment of chromosomal aberration patterns characteristic for bladder cancer. Biochem Biophys Res Commun.

[39] Horst BA, Terrano D, Fang Y, Silvers DN, Busam KJ (2013). 9p21 gene locus in Spitz nevi of older individuals: absence of cytogenetic and immunohistochemical findings associated with malignancy. Hum Pathol.

[40] Muto S, Horie S, Takahashi S, Tomita K, Kitamura T (2000). Genetic and epigenetic alterations in normal bladder epithelium in patients with metachronous bladder cancer. Cancer Res.

[41] Halling KC, Kipp BR (2008). Bladder cancer detection using FISH (UroVysion assay). Adv Anat Pathol.

[42] Sokolova IA, Halling KC, Jenkins RB, Burkhardt HM, Meyer RG, Seelig SA, King W (2000). The development of a multitarget, multicolor fluorescence in situ hybridization assay for the detection of urothelial carcinoma in urine. J Mol Diagn.

[43] Zhivotovsky B, Kroemer G (2004). Apoptosis and genomic instability. Nat Rev Mol Cell Biol.

[44] Baker DJ, Dawlaty MM, Wijshake T, Jeganathan KB, Malureanu L, van Ree JH, Crespo-Diaz R, Reyes S, Seaburg L, Shapiro V, Behfar A, Terzic A, van de Sluis B, Van Deursen JM (2013). Increased expression of BubR1 protects against aneuploidy and cancer and extends healthy lifespan. Nat Cell Biol.

[45] Freedman ND, Silverman DT, Hollenbeck AR, Schatzkin A, Abnet CC (2011). Association between smoking and risk of bladder cancer among men and women. JAMA.

[46] Gandhi NM, Morales A, Lamm DL (2013). Bacillus Calmette-Guerin immunotherapy for genitourinary cancer. BJU Int.

[CR47] The pre-publication history for this paper can be accessed here:http://www.biomedcentral.com/1471-2407/14/854/prepub

